# Yiguanjian decoction inhibits macrophage M1 polarization and attenuates hepatic fibrosis induced by CCl_4_/2-AAF

**DOI:** 10.1080/13880209.2021.1961820

**Published:** 2021-08-23

**Authors:** Ying Xu, Wen Xu, Wei Liu, Gaofeng Chen, Shili Jiang, Jiamei Chen, Xun Jian, Hua Zhang, Ping Liu, Yongping Mu

**Affiliations:** a Shuguang Hospital Affiliated to Shanghai University of Traditional Chinese Medicine (TCM), Shanghai, Pudong District, China; b Institute of Liver Diseases, Shanghai University of TCM, Shanghai, China; c Key Laboratory of Liver and Kidney Disease of the Ministry of Education, Shanghai, China; d Clinical Key Laboratory of TCM of Shanghai, Shanghai, Pudong District, China

**Keywords:** Liver cirrhosis, hepatic progenitor cells, Wnt signalling pathway

## Abstract

**Context:**

Our previous studies indicated that Yiguanjian decoction (YGJ) has an anti-hepatic-fibrosis effect and could regulate macrophage status.

**Objective:**

To elucidate the mechanism of YGJ in regulating macrophages.

**Materials and methods:**

Liver cirrhosis was induced by CCl_4_ for 12 weeks combined with 2-acetylaminofluorene (2-AAF) for the last 4 weeks in male Wistar rats. YGJ (3.56 mg/kg) orally administered in the last 4 weeks, and SORA (1 mg/kg) as control. *In vitro*, RAW264.7 cells were treated with lipopolysaccharides (LPSs) to induce macrophage polarization to the M1 phenotype, and they were co-cultured with WB-F344 cells and allocated to M group, YGJ group (2 μg/mL) and WIF-1 group (1 μg/mL) with untreated cells as control. The differentiation direction of WB-F344 cell line was observed in the presence or absence of YGJ. Pathology, fibrosis-related cytokines, macrophage polarization-related components, and Wnt signalling pathway components were detected.

**Results:**

*In vivo*, the expression levels of α-SMA, Col (1), OV6, SOX9, EpCAM and M1 macrophage-related components (STAT1, IRF3, IRF5, IRF8, SOCS3) significantly decreased in the YGJ group compared with those in the 2-AAF/CCl_4_ group (*p* < 0.01 or 0.05). *In vitro*, the expression levels of M1 macrophage-related components, including STAT1, NF-κB, IRF3, IRF5, and SOCS3, in RAW264.7 cells decreased significantly in the YGJ group compared with those in the M group (*p* < 0.05 or *p* < 0.01). The expression levels of Wnt3A, FZD5, LRP-5/-6, and β-catenin significantly increased in the YGJ group compared with those in the M group (*p* < 0.05 or *p* < 0.01). In addition, the expression levels of Wnt-4/-5A/-5B, and FZD2 significantly decreased in the YGJ group compared with those in the M group (*p* < 0.05 or *p* < 0.01).

**Conclusion:**

This study suggests that the anti-cirrhosis effect of YGJ is associated with its ability to inhibit macrophage M1-polarization, which provides a scientific basis for the clinical application of YGJ.

## Introduction

Liver cirrhosis has emerged as a major contributor to the global health burden (Mokdad et al. 2014; Wang et al. 2016). Kupffer cells (KCs), also known as macrophages of the liver, regulate fibrosis development by modulating pro- and anti-inflammatory pathways (Chen et al. 2013; You et al. 2013). Distinct macrophage subsets express different types of chemokines and surface markers and exhibit diverse functions (Anderson and Mosser 2002; Gordon 2003; Rauh et al. 2005). In the liver, M1 macrophages are induced by pro-inflammatory mediators and produce an abundance of pro-inflammatory cytokines, such as lipopolysaccharides (LPSs). By contrast, M2 macrophages are involved in blocking the inflammatory response and promoting tissue repair (Mosser 2003; Moestrup and Møller 2004; Arnold et al. 2007; Hume 2015). Macrophages also regulate stem cell differentiation by secreting Wnt ligands (Carpino et al. 2016).

Yiguanjian decoction (YGJ) from ‘Liuzhou Yihua’ in the Qing Dynasty of China is a representative recipe for liver cirrhosis with liver-kidney yin deficiency. YGJ consists of six medicinal herbs, namely, Radix rehmanniae (*Rehmannia glutinosa* (Gaetn.) Libosch. ex Fisch. et Mey.), Radix glehniae (*Glehnia littoralis* Fr. Schmidt ex Miq.), Radix ophiopogonis (*Ophiopogon japonicus* (Thunb.) Ker-Gawl.), Fructus lycii (*Lycium barbarum* L.), Radix Angelicae sinensis (*Angelica sinensis* (Oliv.) Diels), and Fructus toosendan (*Melia toosendan* Sieb. et Zucc.) (Reference to pharmacopoeia of the People's Republic of China). Our previous studies indicated that YGJ exerts antifibrotic effects in rodent models by inhibiting hepatocyte apoptosis and hepatic stellate cell (HSC) activation, suppressing angiogenesis, inhibiting the migration of bone marrow cells to the liver, and regulating KC status (Mu et al. 2009; Wang et al. 2012; Lin et al. 2011; Zhou et al. 2015; Xu et al. 2018; Li et al. 2019). However, the mechanisms through which YGJ regulates the state of macrophages remain unclear. In this study, we found that the effect of YGJ against liver fibrosis is related to the inhibition of M1 polarization of macrophages.

## Materials and methods

### Materials

YGJ ingredients (18 g of Rehmanniae radix, 10 g of Glehniae radix, 10 g of Ophiopogonis radix, 12 g of Lycii fructus, 10 g of Radix *Angelicae sinensis*, and 4.5 g of Toosendan fructus) were provided by Shanghai Huayu Herbs Co. Ltd., All herbs were powdered and further extracted by 10 L 20% ethanol twice. Afterward, the extracted liquid was filtered and was subsequently evaporated through rotary vaporation under decompressing, and was vacuum dehydrated to afford extract of YGJ. The process was conducted in Shuguang Hospital affiliated to Shanghai University of Traditional Chinese Medicine (TCM), and the solution was stored at −20 °C. Chemical analysis of YGJ and its fractions by UHPLC-QOrbitrap HRMS. The results have been shown in the Supplementary materials.

Sorafinib (SORA), purchased from Bayer (Leverkusen, Germany), was used as a positive control drug.

Mouse monoclonal antibody against α-smooth muscle actin (α-SMA, Clone 1A4) was obtained from Sigma-Aldrich (St. Louis, MO, USA). Rabbit polyclonal antibody against collagen type I (Col (1) was obtained from ProSci (Poway, CA, USA). Rabbit polyclonal antibodies against Oval Cell Marker (OV6), cytokeratin (CK) 19, signal transducer and activator of transcription (STAT) 5 A, STAT5B, STAT6, interferon regulatory factor (IRF) 3, IRF5, IRF8, frizzled (FZD) 2, and LDL receptor-related protein (LRP) 5 were purchased from Proteintech Group Inc. (Chicago, IL, USA). Rat monoclonal antibody against CD68 and CD163 were obtained from Hycult Biotech (Frontstraat, UDEN, NLD). Rabbit polyclonal antibodies against STAT1, STAT3, and rabbit monoclonal antibodies against nuclear factor kappa-light-chain-enhancer of activated B cells (NF-κB) were purchased from Cell Signalling Technology (Danvers, MA, USA). Rabbit polyclonal antibodies against suppressor of cytokine signalling (SOSC) 2, SOSC3, epithelial cell adhesion molecule (EpCAM), and rabbit monoclonal antibodies against β-catenin were purchased from Abcam (Cambridge, UK). Rabbit polyclonal antibodies against Wnt5A and Wnt5B were purchased from Signalway Antibody LLC (MD, USA). Mouse monoclonal antibody against glyceraldehyde-3-phosphate dehydrogenase (GAPDH) was purchased from Chemicon International (Temecula, CA, USA). Horseradish peroxidase (HRP)-conjugated polyclonal swine anti-rabbit and HRP-conjugated polyclonal rabbit anti-mouse antibodies were purchased from Dako Denmark A/S (Glostrup, Denmark). ECL detection reagent and Hybond-ECL nitrocellulose membranes were purchased from Amersham Pharmacia Biotech (Buckinghamshire, UK). Other reagents were purchased from Wako Pure Chemical Industries, Ltd. (Osaka, Japan) or Sigma Chemical.

### Animals and the rat model of liver fibrosis

Male Wistar rats (aged 7–8 weeks and weighing 160–180 g, *n* = 32) were obtained from the Shanghai Experimental Animal Centre of the Chinese Academy of Sciences (Shanghai, China). The animals were maintained in a constant-temperature environment and supplied with standard laboratory chow and water *ad libitum*. All experimental protocols were approved by the Animal Research Committee of Shanghai University of TCM (No. 20170132).

The rat fibrotic model of progressive cirrhosis was induced by CCl_4_ combined with 2-acetylaminofluorene (2-AAF) (Chen et al. 2015). Rats were subcutaneously injected with 50% CCl_4_-olive oil solution (2 mL/kg) twice a week for 8 weeks. From the ninth week, the concentration of CCl_4_-olive oil solution was adjusted to 30% (2 mL/kg), and all model rats were treated with 2-AAF (10 mg/kg/d) via oral administration once a day and continuously administered for 4 weeks to maintain the progress of hepatic fibrosis. On the first day of week 9, the fibrotic rats were randomly divided into groups (*n* = 8): 2-AAF/CCl_4_, 2-AAF/CCl_4_ + YGJ (YGJ), and 2-AAF/CCl_4_ + SORA (SORA). YGJ and SORA were orally administered at dosages of 3.56 and 1.0 mg/kg, respectively, once per day for 4 weeks. Naive, untreated rats (N; *n* = 8) received an equal amount of subcutaneously administered olive oil and orally administered volume of solvent. At the end of the animal experiment, all rats were euthanized with pentobarbital sodium at a dose of 60 mg/kg, and blood and hepatic tissue samples were obtained.

### Serum chemistry

Serum alanine aminotransferase (ALT) and aspartate aminotransferase (AST) were detected by the experimental centre of Shanghai University of TCM.

### Histochemical and immunohistochemical analyses

Paraformaldehyde-fixed (4%) specimens were cut into 4 μm sections and stained with haematoxylin and eosin (H&E) or Sirius Red.

Immunostaining was performed according to a previously published method (Zhang et al. 2016). Briefly, sections were deparaffinized, washed, and preincubated in blocking solution, followed by incubation with anti-α-SMA (1:200), anti-Col (1) (1:200), anti-CD68 (1:200), anti-CD163 (1:200), anti-OV6 (1:40), anti-CK7 (1:200), and anti-CK19 (1:200). The sections were incubated with horseradish peroxidase (HRP)-conjugated secondary antibodies (1:1000), washed, stained with 3,3′-diaminobenzidine (DAB), and counterstained with haematoxylin. A Leica SCN 400 microscope was used to scan the stained slides. For immunofluorescent staining, Alexa Fluor 488 (1:2000) and cyanine 3 (1:2000) secondary antibodies (Jackson ImmunoResearch, West Grove, PA, USA) were used with counterstaining. Images were acquired using a laser scanning confocal microscope (FV10i, Olympus, Japan).

### Immunoblot analysis

Protein levels were assessed by immunoblot analysis as previously described (Zhang et al. 2016). Liver tissue was lysed with RIPA buffer containing 50 mM Tris-HCl (pH 7.2), 150 mM NaCl, 1% nonyl-phenoxypolyethoxylethanol (NP-40), 0.1% sodium dodecyl sulphate (SDS), 1 mM ethylenediaminetetraacetic acid, and 1 mM phenylmethylsulfonyl fluoride and then homogenized in ice-cold water. After centrifugation for 10 min at 4 °C and 12,000 rpm, the protein concentration of the supernatant was determined using the Bio-Rad Dc Protein Assay Reagent (Bio-Rad, Hercules, CA, USA). The proteins were electrophoretically decomposed on a 10 or 12% SDS polyacrylamide gel and transferred to Hybond-ECL nitrocellulose membranes. The membranes were blocked by 5% non-fat dry milk in Tris-buffered saline containing 0.1% Tween-20. Then, the membranes were incubated with primary antibodies overnight at 4 °C, followed by incubation with HRP-conjugated secondary antibodies at room temperature for 1 h. The following dilutions of primary antibodies were used: 1:1000 for α-SMA, Col(1), NF-κB, STAT1, IRF5, SOSC3, CK19, FZD2, LRP5, EpCAM, Wnt5A and Wnt5B; 1:2000 for IRF3 and IRF8; 1:5000 for β-catenin; and 1:30,000 for GAPDH. Immune complexes were visualized using SuperSignal West Pico Chemiluminescent Substrate (ECL, Pierce, Rockford, IL, USA). Finally, protein band intensity was determined by scanning video densitometry.

### RNA preparation and quantitative real-time reverse transcription polymerase chain reaction (RT-PCR)

Total RNA was extracted from the liver tissue using a total RNA purification kit (Lot. 250800; TOYOBO, Osaka, Japan). RNA was reverse transcribed to cDNA, and gene expression was measured using a SYBR Green Real-time PCR Master Mix (Lot. 411900) (TOYOBO) and the ViiA 7 Real-Time PCR System (ABI, American). Primers and oligonucleotide probes were designed using Primer Express (Takara Chemical) and are listed in Table 1. The mRNA expression of TNF-α, TGF-β-1, α-SMA, Col (1), Hep, CD68, CD163, OV6, SOX9, EpCAM, CK19, STAT1, NF-κB, IRF3, IRF5, IRF8, SOSC3, Wnt-4/Wnt-5A/Wnt-5B/Wnt-11, β-catenin, FZD-2/FZD-3/FZD-6, and GAPDH was quantified using quantitative RT-PCR performed on five samples (*in vivo*) or on three samples (*in vitro*) in both the experimental and control groups. Individual gene expression was normalized to GAPDH expression levels. The parameters for the One-Step SYBR RT-PCR (Perfect Real Time) were as follows: an initial step of 15 min at 42 °C, 2 min at 95 °C, 40 amplification cycles of denaturation at 95 °C for 15 s, and annealing and extension at 60 °C for 1 min.

**Table 1.** Primer pairs and probes used for Real-time PCR.

Gene		Primer sequence	Gene		Primer sequence
*α-SMA*	Forward	AATGGCTCTGGGCTCTGTAA	*Wnt 1*	Forward	GGGGAGCAACCAAAGTCG
	Reverse	TCTCTTGCTCTGGGCTTCAT		Reverse	TGGAGGAGGCTATGTTCACG
*Col1*	Forward	TGACTGGAAGAGCGGAGAGT	*Wnt 3 A*	Forward	TCCGACTCTTGGCAGAACTT
	Reverse	GACGGCTGAGTAGGGAACAC		Reverse	AATGGAATAGGTCCCGAACA
*TGF-β1*	Forward	ATTCCTGGCGTTACCTTGG	*Wnt 4*	Forward	GGCACTCATGAACCTTCACAACA
	Reverse	AGCCCTGTATTCCGTCTCCT		Reverse	CTTTACCTCACAGGAGCCTGACAC
*TNF-α*	Forward	GACGTGGAACTGGCAGAAGAG	*Wnt 5 A*	Forward	GCGCTGCTGGAGTGGTAAAT
	Reverse	TTGGTGGTTTGTGAGTGTGAG		Reverse	AGCCAGTCCCGAGGTAAGTC
*CD68*	Forward	GGACCCACAACTGTCACTCAT	*Wnt 5B*	Forward	CGAGCCCTCATGAACTTACAGAAC
	Reverse	AAGCCCCACTTTAGCTTTACC		Reverse	GGAGACTCCGTGACATTTGCAG
*CD163*	Forward	TGGGATCGCCGTGACGCTTC	*Wnt 8 A*	Forward	CCTGGGAGCGGTGGAACT
	Reverse	CAGCGACTGCCTCCACCGAC		Reverse	CCTGGTGTGGGTTGAAAACTG
*OV6*	Forward	GATGCTGGACACAAACTCAACT	*Wnt 8B*	Forward	AAGGCTTACCTGGTCTACTC
	Reverse	GCC ACA ACAGGA ATCTCTCC		Reverse	CAGAGCTGATGGCGTGCACA
*SOX9*	Forward	GAA AGA CCA CCCCGATTACAAG	*Wnt 10B*	Forward	CCTCAAGCGCGGTTTCC
	Reverse	AAGATGGCGTTAGGA GAGATGTG		Reverse	CAGCAGCCAGCATGGAGAA
*EpCAM*	Forward	TGTGGACATAGCTGATGTGGCTTA C	*Wnt 11*	Forward	TTGACCTGGAGAGAGGTACAC
	Reverse	CACCCTCAGGTCCATGCTCTT A		Reverse	GTCAGGGGAGCTCTGTAGATA
*CK19*	Forward	TATCTGGATCTGCGTAGTGTGG	*Frizzled 1*	Forward	GGGAATGCAGTCACCAGTACCA
	Reverse	ATACAA AACCAA ACTGGGGATG		Reverse	CCAGACCCATAGCAGGTTCCA
*Hep*	Forward	TAGCAGAGATGAGCCGTGTG	*Frizzled 2*	Forwa rd	ACTGCAAGAGCCTAGCCAT CC
	Reverse	GCTTTGAGGCAGGCGTATT		Reverse	ATCCAGAAGCCCGACGTGA
*STAT1*	Forward	GTCCTCTTCCAGCAGCTCATTCAG	*Frizzled 3*	Forward	ACACATGGCACCAGCATGAAC
	Reverse	ACCAACAGTCTCAGCTTCACAGTG		Reverse	CCATGCGAAGGCCAAGACTAA
*IRF3*	Forward	GGCTTACGACAGGACGCACAG	*Frizzled 4*	Forward	GACAACTTTCACGCCGCTCA
	Reverse	CAGGTTGACAGGTCTGGCTTATCC		Reverse	TTCAGGACTGGTTCACATCGTCTC
*IRF5*	Forward	GCTCCATCACATCTGGCAGTCC	*Frizzled 5*	Forward	CGAGAGCACAGCCACATTCACTA
	Reverse	GGAAGTCCAAGTCAGCCACCATC		Reverse	GAGCTGGCCATGCCAAAGA
*IRF8*	Forward	TGGACATTTCCGAGCCATACAA	*Frizzled 6*	Forward	CAGCAGCGTCCAACTCCAAG
	Reverse	CGATCTCTGAACGGCCACAC		Reverse	TGCACTCCATCAGGCCAGTC
*NF-κB*	Forward	GATGGGACGACACCTCTACACATA	*LRP 5*	Forward	GACATTTACTGGCCCAATGG
	Reverse	CCCAAGAGTCGTCCAGGTCA		Reverse	CTGCCCTCCACCACCTTCT
*SOSC3*	Forward	TCAACGGTCACCTGGACTCCTA	*LRP 6*	Forward	TCTCCGGCGAATTGAAAG
	Reverse	GGTCCAGGAACTCCCGAATG		Reverse	GAGTCTTCTAGCACGATCCTGT
*β-catenin*	Forward	GTCTGAGGACAAGCCACAGGACTAC	*GAPDH*	Forward	AAGGTCATCCATGACAACTTTGGC
	Reverse	AATGTCCAGTCCGAGATCAGCA		Reverse	ACAGTCTTCTGGGTGGCAGTGAT

### WB-F344 cell line and RAW264.7 cell line Co-culture

WB-F344 cell line (a rat hepatic progenitor cell line) (Tsao et al. 1984) and RAW264.7 cells (a macrophage-like, Abelson leukaemia virus-transformed cell line) were purchased from Shanghai Institutes for Biological Sciences. WB-F344 cells were cultured in DMEM medium containing 10% foetal bovine serum (FCS), 2 mM glutamine, and penicillin/streptomycin (100 mg/mL). RAW264.7 cells were polarized to M1 phenotype after stimulation with 100 ng/mL of LPS for 8 h (5% CO_2_-in-air atmosphere in DMEM supplemented with 10% FCS) (Ploeger et al. 2013); then, RAW264.7 cells were co-cultured with WB-F344 cells in transwell chambers. WB-F344 cells (2 × 10^4^ cells) were seeded in the upper part of the transwell chamber, and RAW 264.7 cells (4 × 10^4^) were seeded in the lower part. The medium was changed every 24 h for 7 days. The groupings were as follows: WB-F344 cells co-cultured with inactivated RAW264.7 cells (N); WB-F344 cells co-cultured with LPS (100 ng/mL)-activated RAW264.7 cells (referred to as LPS-RAW264.7) (M); WB-F344 cells co-cultured with LPS-RAW264.7 treated with refined YGJ (YGJ, 2 μg/mL); and WB-F344 cells co-cultured with LPS-RAW264.7 treated with Wnt inhibitory factor-1 (WIF-1, 1 μg/mL).

### Statistical analysis

All data in the experiment are expressed as mean ± standard deviation (SD). SPSS 17.0 was used for statistical analysis, and comparisons between two groups were made using an unpaired *t*-test. For comparisons between multiple groups, one-way ANOVA was used. *p* < 0.05 was considered statistically significant.

## Results

### YGJ inhibits the activation of hepatic stellate cells and the progression of liver fibrosis

**Figure 1.**
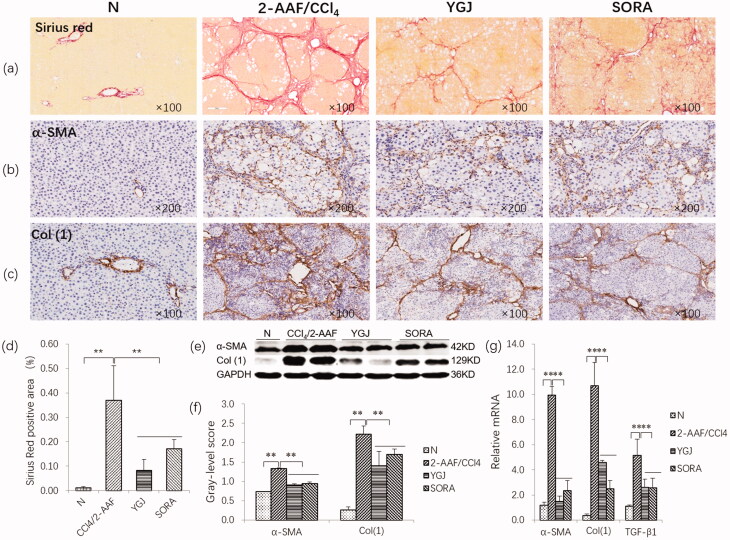
YGJ inhibits the activation of hepatic stellate cells and fibrosis. (a) Sirius red staining (×100). (b) α-SMA immunostaining (×200). (c) Col (1) immunostaining (×200). (d) Sirius Red-positive region. (e) α-SMA and Col(1) protein bands were depicted in immunoblots, and (f) the histogram is a densitometric analysis of the protein bands (*n* = 5 per group). (g) α-SMA, Col(1) and TGF-β mRNA expressions were measured by RT-PCR and normalized to GAPDH mRNA (*n* = 5 per group). **p < 0.01. *N*: untreated group (control); 2-AAF/CCl_4_: 2-acetylaminofluorene/carbon tetrachloride-treated group; YGJ: 2-AAF/CCl_4_ + Yiguanjian decoction-treated group; SORA: 2-AAF/CCl_4_ + sorafenib-treated group.

Sirius Red staining showed that the collagen in the bridge connecting the central veins and neighbouring portal areas was continually increased, and pseudonodules were formed in the 2-AAF/CCl_4_ group; they were markedly attenuated in the YGJ group compared with those in the 2-AAF/CCl_4_ group (Figure 1(a)). In addition, the positive area of Sirius Red staining was increased significantly in the 2-AAF/CCl_4_ group compared with that in the N group (*p* < 0.01) but was decreased significantly in the YGJ group compared with that in the 2-AAF/CCl_4_ group (*p* < 0.01) (Figure 1(d)).

Immunostaining showed that α-SMA and Col (1) expression was markedly increased in the 2-AAF/CCl_4_ group, while these pathological changes were clearly alleviated in the YGJ group (Figure 1(b,c)). Immunoblot analysis showed that the protein expression levels of α-SMA and Col (1) were significantly increased in the 2-AAF/CCl_4_ group compared with those in the N group (*p* < 0.01) but were significantly decreased in the YGJ group compared with those in the 2-AAF/CCl_4_ group (*p* < 0.01) (Figure 1(e,f)). The mRNA expression levels of α-SMA, Col (1), and TGF-β1 were increased significantly in the 2-AAF/CCl_4_ group compared with those in the N group (*p* < 0.01) but were decreased significantly in the YGJ group compared with those in the 2-AAF/CCl_4_ group (*p* < 0.01) (Figure 1(g)).

### YGJ inhibits the inflammation response and bile duct reaction in the liver

**Figure 2.**
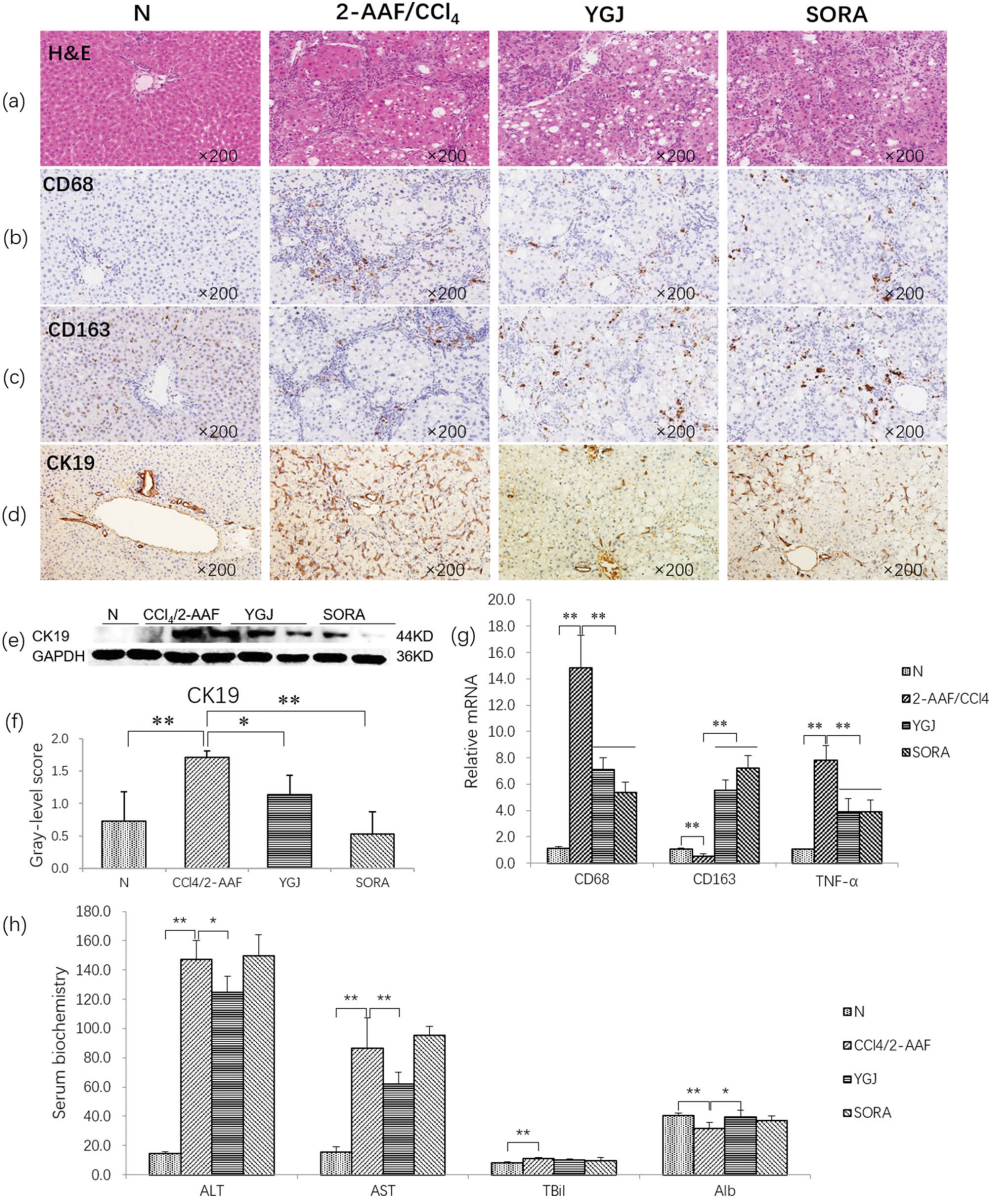
YGJ inhibits liver inflammation and bile duct reaction. (a) H&E staining (×200). (b) CD68 immunostaining (×200). (c) CD163 immunostaining (×200). (d) CK19 immunostaining (×200). (e) CK19 protein bands were depicted in the immunoblot images, and (f) the densitometric quantification of the protein bands presented as a histogram (*n* = 5 per group). (g) CD68, CD163, and TNF-α mRNA expressions were measured by RT-PCR and normalized to GAPDH mRNA (*n* = 5 per group). (h) Serum levels of alanine aminotransferase and aspartate aminotransferase. *p < 0.05 and **p < 0.01. N: untreated group (control); 2-AAF/CCl_4_: 2-acetylaminofluorene/carbon tetrachloride-treated group; YGJ: 2-AAF/CCl_4_ + Yiguanjian decoction-treated group; SORA: 2-AAF/CCl_4_ + sorafenib-treated group.

H&E staining showed extensive hepatocyte steatosis and hepatic lobular structural disorders in the 2-AAF/CCl_4_ group. However, these pathological changes were suppressed by YGJ treatment (Figure 2(a)). Immunostaining showed that CD68 (a marker of M1 macrophage) expression was markedly increased, but CD163 (a marker of M2 macrophage) expression was clearly decreased in the 2-AAF/CCl_4_ group. By contrast, CD68 expression was markedly decreased and CD163 was clearly increased after YGJ treatment (Figure 2(b,c)). The mRNA expression levels of CD68 and TNF-α (secreted by M1 macrophages) were increased significantly in the 2-AAF/CCl_4_ group compared with those in the N group (*p* < 0.01). However, the expression levels were reduced significantly in the YGJ group compared with those in the 2-AAF/CCl_4_ group (*p* < 0.01) (Figure 2(g)). Conversely, the mRNA expression of CD163 was decreased significantly in the 2-AAF/CCl_4_ group compared with that in the N group (*p* < 0.01) but was increased significantly in the YGJ group compared with that in the 2-AAF/CCl_4_ group (*p* < 0.01) (Figure 2(g)). Immunostaining showed that CK19 expression was markedly increased in the 2-AAF/CCl_4_ group compared with that in the N group but was decreased in the YGJ group compared with that in the 2-AAF/CCl_4_ group (Figure 2(d)). Consistent with immunostaining, immunoblot analysis also showed that the protein expression level of CK19 was significantly increased in the 2-AAF/CCl_4_ group compared with that in the N group (*p* < 0.01). However, it was significantly decreased in the YGJ group compared with that in the 2-AAF/CCl_4_ group (*p* < 0.01) (Figure 2(e,f)).

In addition, serum liver function assay showed that in the 2-AAF/CCl_4_ group, ALT and AST activation was increased significantly compared with that in the N group (*p* < 0.01). However, it was decreased significantly in the YGJ group compared with that in the 2-AAF/CCl_4_ group (*p* < 0.01) (Figure 2(h)).

### YGJ inhibits the macrophage M1 polarization and the activation of non-canonical wnt signalling pathway

**Figure 3.**
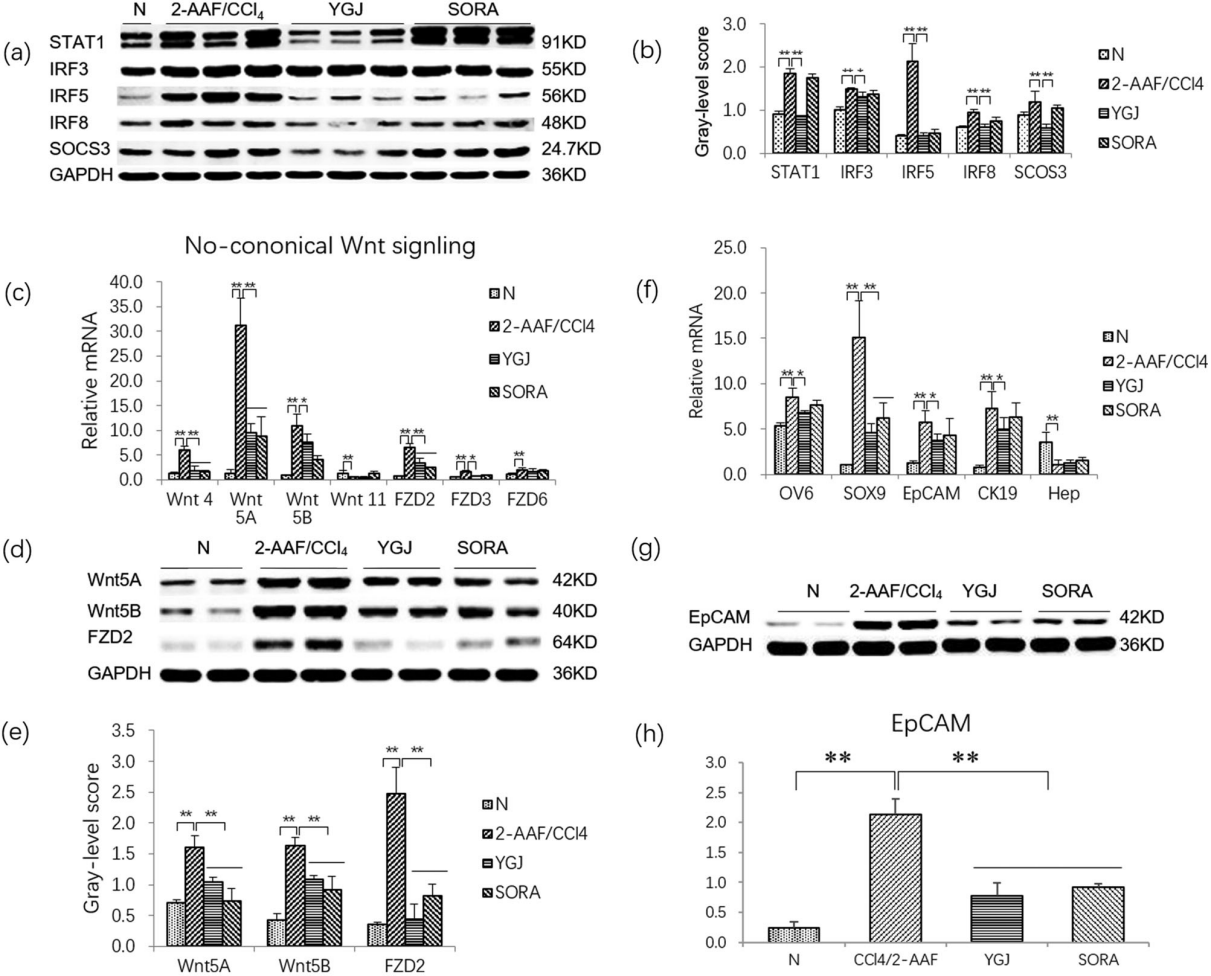
YGJ inhibits the activation of M1 macrophages and non-canonical Wnt signalling pathways and inhibits the differentiation of hepatic progenitor cells into myofibroblasts *in vivo*. (a) STAT1, IRF3, IRF5, IRF8, and SOCS3 protein bands were depicted in the immunoblot images, and (b) the densitometric quantification of the protein bands presented as a histogram (*n* = 5 per group). (c) The mRNA expressions of Wnt-4, -5 A, -5B, FZD-2, -3, and -6 in liver were measured by RT-PCR and normalized to GAPDH mRNA (*n* = 5 per group). (d) Wnt5A, Wnt5B, and FZD2 protein bands were depicted in the immunoblot images, and (e) the densitometric quantification of the protein bands presented as a histogram (*n* = 5 per group). (f) OV6, SOX9, EpCAM, CK19, and Hep mRNA expressions were measured by RT-PCR and normalized to GAPDH mRNA (*n* = 5 per group). (g) EpCAM protein bands were depicted in the immunoblot images, and (h) the densitometric quantification of the protein bands presented as a histogram (*n* = 5 per group). *p < 0.05 and **p < 0.01. N: untreated group (control); 2-AAF/CCl_4_: 2-acetylaminofluorene/carbon tetrachloride-treated group; YGJ: 2-AAF/CCl_4_ + Yiguanjian decoction-treated group; SORA: 2-AAF/CCl_4_ + sorafenib-treated group.

Figures 3(a,b) show that the protein levels of M1 macrophage-related components, including STAT1, IRF3, IRF5, IRF8, and SOCS3, were increased significantly in the 2-AAF/CCl_4_ group compared with those in the N group (*p* < 0.05 or *p* < 0.01). However, they were decreased significantly in the YGJ group compared with those in the 2-AAF/CCl_4_ group (*p* < 0.05 or *p* < 0.01), suggesting that YGJ inhibits macrophage polarization into M1 phenotypes.

Given that macrophages are closely related to the activation of the Wnt signalling pathway, we further examined the mRNA expression levels of key molecules in this pathway. The mRNA levels of non-canonical Wnt-pathway components, including Wnt-4/-5A/-5B, and FZD-2/-3/-6, were increased significantly in the 2-AAF/CCl_4_ group compared with those in the N group (*p* < 0.01). The mRNA expression levels of Wnt-4/-5A/-5B and FZD-2/-3 were decreased significantly in the YGJ group compared with those in the 2-AAF/CCl_4_ group (*p* < 0.05 or *p* < 0.01) (Figure 3(c)). The protein expression levels of Wnt-5A/-5B and FZD2 were increased significantly in the 2-AAF/CCl_4_ group compared with those in the N group (*p* < 0.01) but were significantly decreased in the YGJ group compared with those in the 2-AAF/CCl_4_ group (*p* < 0.01) (Figure 3(d,e)). These results suggested that YGJ can inhibit the activation of non-canonical Wnt signalling *in vivo*.

As we previously reported in 2-AAF/CCl_4_ models, hepatic progenitor cells are activated and differentiated into myofibroblasts (Chen et al. 2015). In the present study, the mRNA expression levels of the hepatic progenitor cell markers OV6, SOX9, EpCAM, and the cholangiocyte marker CK19 were increased significantly in the 2-AAF/CCl_4_ group compared with those in the N group (*p* < 0.01); they were reduced significantly after YGJ treatment (*p* < 0.05 or *p* < 0.01). The protein expression level of EpCAM was increased significantly in the 2-AAF/CCl_4_ group compared with that in the N group (*p* < 0.01) but was significantly decreased in the YGJ group compared with that in the 2-AAF/CCl_4_ group (*p* < 0.01) (Figures 3(g,h)). The mRNA expression of Hep (a hepatocyte marker) was decreased significantly in the 2-AAF/CCl_4_ group compared with that in the N group (*p* < 0.01), but no significant change was noted after YGJ treatment (*p* > 0.05) (Figure 3(f)). This result suggests that YGJ can effectively inhibit the activation of hepatic progenitor cells and bile duct reaction, but the effects on liver regeneration may be limited in the 2-AAF/CCl_4_ model.

### YGJ can inhibit the differentiation of WB-F344 cells induced by M1 polarized macrophages

**Figure 4.**
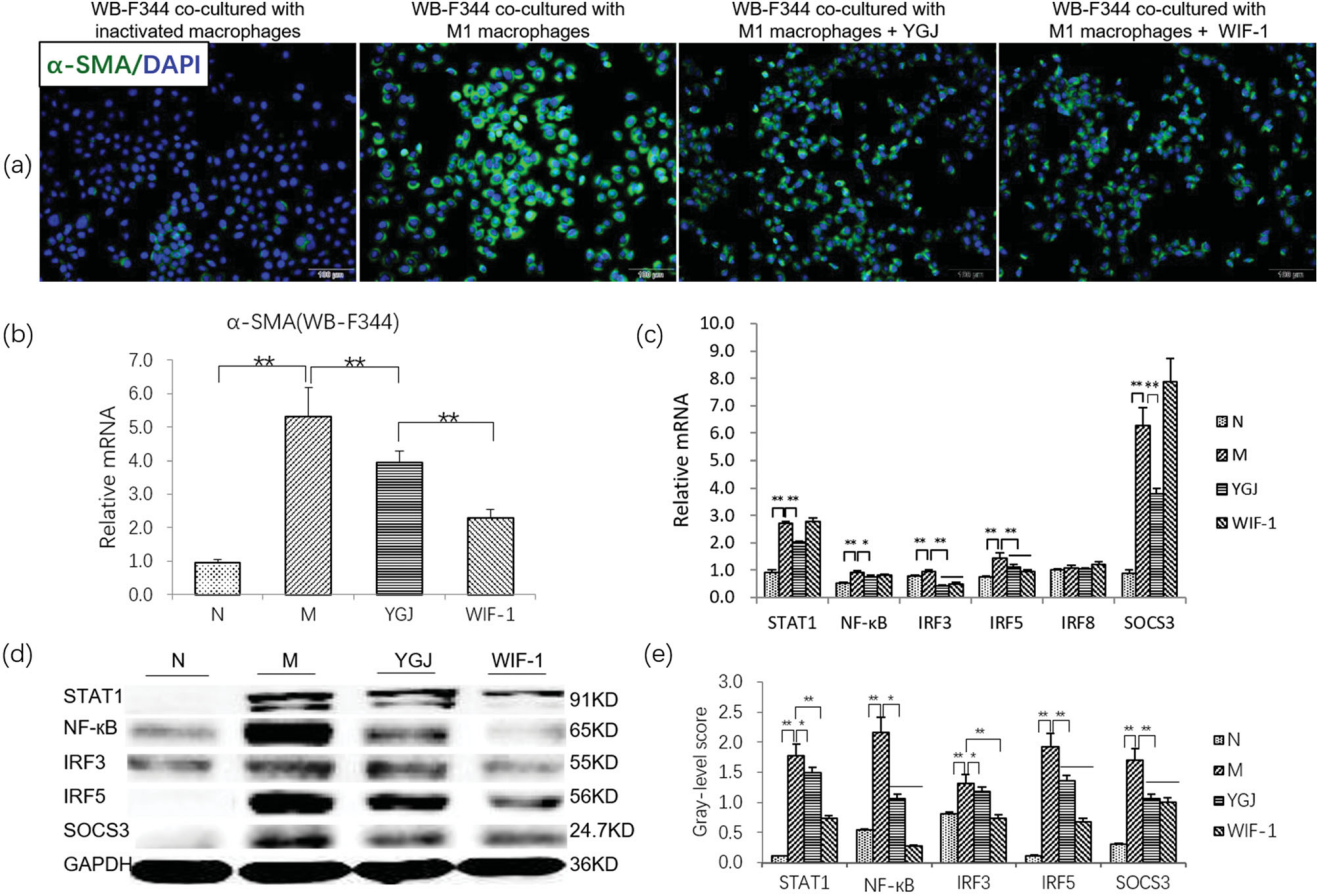
YGJ inhibits the differentiation of WB-F344 cells into myofibroblasts through inhibiting the activation of M1 macrophages. (a) α-SMA immunofluorescent staining (green) in WB-F344 cells (×200) (*the presented in vitro experiments were conducted in the same batch, the normal and model groups used the same pictures as published articles. The figures of WB-F344 co-cultured with inactivated macrophages and WB-F344 co-cultured with M1 macrophages were reused form a previous study* [Ying Xu et al. 2018] *and are reproduced with permission here*). (b) α-SMA mRNA expression in WB-F344 cells was measured by RT-PCR and normalized to GAPDH mRNA (*n* = 3 per group). (c) STAT1, NF-κB, IRF3, IRF5, IRF8, and SOCS3 mRNA expressions in RAW264.7 cells were measured by RT-PCR and normalized to GAPDH mRNA (*n* = 3 per group); (d) STAT1, NF-κB, IRF3, IRF5, and SOCS3 protein bands were depicted in the immunoblot images, and (e) the densitometric quantification of the protein bands presented as a histogram (*n* = 3 per group). *p < 0.05 and **p < 0.01. N: WB-F344 cells co-cultured with inactivated RAW264.7 cells; M: WB-F344 cells co-cultured with LPS (100 ng/mL)-activated RAW264.7 cells (referred to as LPS-RAW264.7); YGJ: WB-F344 cells co-cultured with LPS-RAW264.7 treated with Yiguanjian decoction; WIF-1: WB-F344 cells co-cultured with LPS-RAW264.7 treated with Wnt inhibitory factor-1.

Immunofluorescence staining showed that the protein expression of α-SMA in WB-F344 cells was markedly increased in the M group, but this increase was suppressed by adding YGJ or WIF-1 (a blocker of non-canonical Wnt pathway) (Figure 4(a)) (The figures of WB-F344 co-cultured with inactivated macrophages and WB-F344 co-cultured with M1 macrophages were reused from a previous study, and permission from the publisher was obtained). The mRNA level of α-SMA in WB-F344 cells was increased significantly in the M group compared with that in the N group (*p* < 0.01) but was decreased significantly in the YGJ and WIF-1 groups compared with that in the M group (*p* < 0.01) (Figure 4(b)).

Figure 4(c) shows that the mRNA expression levels of M1 macrophage-related components, including STAT1, NF-κB, IRF3, IRF5, and SOCS3, in RAW264.7 cells were increased significantly in the M group compared with that in the N group (*p* < 0.01); however, the above indicators were decreased significantly in the YGJ group compared with that in the M group (*p* < 0.05 or *p* < 0.01). In addition, the protein expression levels of STAT1, NF-κB, IRF3, IRF5, and SOCS3 in RAW264.7 cells were increased significantly in the M group compared with those in the N group (*p* < 0.01). However, they were significantly decreased in the YGJ group compared with those in the M group (*p* < 0.05 or *p* < 0.01) (Figure 4(d,e)). Thus, YGJ may suppress macrophage polarization into the M1 phenotype and inhibit the differentiation of WB-F344 cells into myofibroblasts *in vitro*.

### YGJ inhibition of WB-F344 cell differentiation into myofibroblasts is related to the regulation of wnt signalling activation in vitro

**Figure 5.**
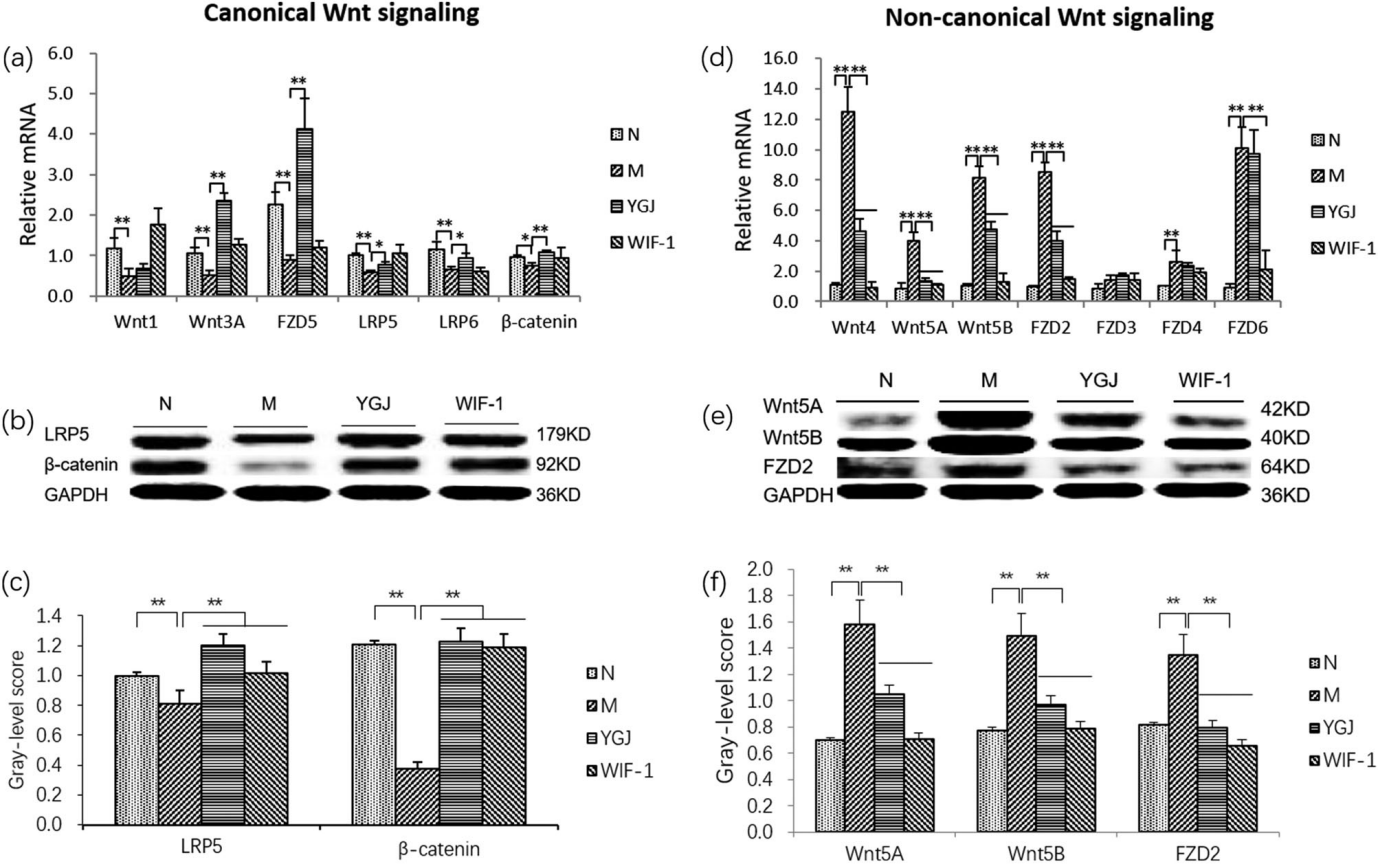
YGJ inhibits the differentiation of WB-F344 cells into myofibroblasts is related to the regulation of Wnt signalling activation. (a) Wnt-1, -3 A, FZD5, LRP-5, -6, and β-Catenin mRNA expressions in WB-F344 cells were measured by RT-PCR and normalized to GAPDH mRNA (*n* = 3 per group); (b) LRP-5 and β-Catenin protein bands were depicted in the immunoblot images, and (c) the densitometric quantification of the protein bands presented as a histogram (*n* = 3 per group). (d) Wnt-4, -5 A, -5B, FZD-2, -3, -4, and -6 mRNA expressions in WB-F344 cells were measured by RT-PCR and normalized to GAPDH mRNA (*n* = 3 per group); (e) Wnt -5 A, -5B, and FZD-2 protein bands were depicted in the immunoblot images, and (f) the densitometric quantification of the protein bands presented as a histogram (*n* = 3 per group). *p < 0.05 and **p < 0.01. N: WB-F344 cells co-cultured with inactivated RAW264.7 cells; M: WB-F344 cells co-cultured with LPS (100 ng/mL)-activated RAW264.7 cells (referred to as LPS-RAW264.7); YGJ: WB-F344 cells co-cultured with LPS-RAW264.7 treated with Yiguanjian decoction; WIF-1: WB-F344 cells co-cultured with LPS-RAW264.7 treated with Wnt inhibitory factor-1.

Furthermore, the mRNA expression levels of canonical Wnt-signalling components Wnt-1/-3A, FZD5, LRP-5/-6, and β-catenin in WB-F344 cells were decreased significantly in the M group compared with those in the N group (*P* < 0.01), while the mRNA expression levels of Wnt 3 A, FZD5, LRP-5/-6, and β-catenin were significantly increased in the YGJ group compared with those in the M group (*p* < 0.05 or *p* < 0.01) (Figure 5(a)). In addition, the protein expression levels of LRP5 and β-catenin were decreased significantly in the M group compared with those in the N group (*p* < 0.01) but were significantly increased in the YGJ group compared with that in the M group (*p* < 0.01) (Figure 5(b,c)).

However, the mRNA expression levels of non-canonical Wnt-signalling components, including Wnt-4/-5A/-5B and FZD-2/-4/-6 in WB-F344 cells were increased significantly in the M group compared with those in the N group (*p* < 0.01). The mRNA levels of Wnt-4/-5A/-5B, and FZD 2 were significantly decreased in the YGJ group compared with those in the M group (*p* < 0.05 or *p* < 0.01) (Figure 5(d)). The protein expression levels of Wnt-5A/-5B, and FZD 2 were increased significantly in the M group compared with those in the N group (*p* < 0.01), while they were significantly decreased in the YGJ group compared with that in the M group (*p* < 0.01) (Figure 5(e,f)). Thus, the mechanism of YGJ regulation of the Wnt signalling pathway in WB-F344 cells may be through suppression of macrophage polarization into M1 *in vitro*.

## Discussion

Our previous study showed that in the rat model of progressive cirrhosis induced by 2-AAF/CCl_4_, endogenous hepatic progenitor cells differentiated into myofibroblasts and promoted fibrosis. Blockade of the Wnt signalling pathway inhibited TGF-β1-induced differentiation of hepatic progenitor cells into myofibroblasts *in vitro* (Chen et al. 2015).

In the present study, YGJ inhibited the liver inflammation response, activation of HSCs, and the progression of liver cirrhosis induced by 2-AAF/CCl_4_. The expression levels of OV6, SOX9, EpCAM, and CK19 were significantly decreased in the liver after YGJ treatment, suggesting that the anti-liver fibrosis effect of YGJ is not only related to inhibiting HSC activation but also to inhibiting liver progenitor cell activation and bile duct action, and these effects are closely related to macrophage polarization.

### YGJ inhibits macrophage M1 polarization

Macrophages are keen sensors of tissue homeostasis and can rapidly switch between pro- and anti-inflammatory regulatory functions in response to different perturbations in their microenvironment (Xu et al. 2018). These different functional phenotypes of macrophages are generally classified into two main types: those conditioned by pro-inflammatory stimuli, such as interferon (IFN)-γ, also referred to as classically activated or M1 macrophages, and those polarized by anti-inflammatory cytokines, such as interleukin (IL)-4 and IL-13, also known as alternatively activated or M2 macrophages (Mosser and Edwards 2008; Martinez et al. 2009; Murray et al. 2014).

Meijer et al. (2000) reported that Kupffer cell depletion by liposome-encapsulated dichloromethylene-diphosphonate abolishes the hepatic synthesis of IL-6 and IL-10 mRNA and decreases the hepatic expression of TNF-α, hepatocyte growth factor, and TGF-β1 mRNA after partial hepatectomy. A recent study found that Nogo-B can promote macrophage polarization into the M1 phenotype and further promote the progression of alcoholic liver disease. By contrast, Nogo-B deletion can promote macrophage polarization into the M2 phenotype, thus promoting recovery from alcoholic liver disease (Park et al. 2017), suggesting that KCs are closely related to liver regeneration and damage repair.

In the present study, the expression levels of CD68 and TNF-α mRNA were significantly increased, whereas that of CD163 mRNA was significantly decreased in the liver tissue. These results suggested an imbalance between pro-inflammatory and anti-inflammatory macrophages in this model. In addition, the protein levels of STAT1, IRF3, IRF5, IRF8, and SOCS3 (proteins associated with polarization into M1 macrophages) were significantly increased in the 2-AAF/CCl_4_ group but were significantly decreased after YGJ treatment, suggesting that YGJ has a regulatory effect on the M1 macrophages of liver.

### YGJ regulates the crosstalk between macrophages and hepatic progenitor cells mediated by Wnt signalling pathway

The proliferation and differentiation of hepatic progenitor cells are regulated by various signalling pathways, such as Wnt, Hedgehog, and Notch, especially the Wnt signalling pathway, which is involved in the regulation of all stem cell populations (Reya and Clevers 2005). Wnt ligands (e.g., Wnt1-11) are mainly derived from macrophages and play an important role in tissue regeneration and repair (Wynn et al. 2013). Saha et al. (2016) reported that Wnt ligands derived from the extracellular vesicles of macrophages protect intestinal stem cells and improve the survival rate of intestinal stem cells after radiation injury. They also found that Porcupine (PORCN) is indispensable in the secretion mechanism of Wnt core ligands. The PORCN inhibitor Wnt-C59 abolished the Wnt-secretion function of macrophages and weakened its protective effect on intestinal stem cells, providing direct evidence for Wnts derived from macrophages.

As an important part of the stem cell microenvironment, macrophages have received widespread attention in the field of liver regeneration in recent years (Aurora and Olson 2014). The differentiation of hepatic progenitor cells into hepatocytes is mainly regulated by the classical Wnt/β-catenin signalling pathway and may be related to Wnt3A release by M2 macrophages after phagocytosis of apoptotic hepatocyte fragments and activation of the Wnt/β-catenin signalling pathway in hepatic progenitor cells (Boulter et al. 2012). Wnt3A also promotes IL-4 or TGF-β1-induced macrophage polarization into the M2 phenotype (Feng et al. 2018). The differentiation of hepatic progenitor cells into myofibroblasts may be regulated by TNF-α release by M1 macrophages to activate non-canonical Wnt signalling pathways, such as Wnt5/FZD2 (Jiang et al. 2006). A clinical study has shown that pro-inflammatory macrophages dominate the macrophage population in children with non-alcoholic fatty liver disease, and docosahexaenoic acid treatment can polarize these macrophages into anti-inflammatory phenotypes and upregulate Wnt3A expression. Thus, β-catenin phosphorylation is promoted in hepatic progenitor cells, which then differentiate into hepatocytes (Carpino et al. 2016). Collectively, these findings suggested that Wnt signalling mediated the crosstalk between macrophages and hepatic progenitor cells.

Our previous study showed that the non-canonical Wnt signalling pathway is significantly activated in the rat model of cirrhosis induced by 2-AAF/CCl_4_. *In vitro*, TGF-β1 induced WB-F344 cells to differentiate into myofibroblasts, and the non-canonical Wnt signalling pathway is significantly activated. Wnt signalling blocker WIF-1 inhibits this pathological process (Xu et al. 2018). In the current study, we found that in the components of the non-canonical Wnt signalling pathway, including Wnt-4/-5A/-5B, FZD-2/-3 expression levels were significantly reduced after YGJ treatment. As such, YGJ may have a regulatory effect on the activation of Wnt signalling in liver fibrosis induced by 2-AAF/CCl_4_ in rats.

We used LPS to polarize macrophages into the M1 phenotype *in vitro* and then co-cultured with WB-F344 cells and treated with YGJ or WIF-1. The expression of α-SMA mRNA in WB-F344 cells was significantly increased when the two cell types were co-cultured, and YGJ significantly reduced its expression. Thus, M1 macrophages may have promoted the differentiation of hepatic progenitor cells into myofibroblasts, while YGJ inhibited this pathological process.

The protein levels of molecules related to macrophage polarization to M1, such as NF-ĸB, IRF3, IRF5, STAT1, and SOCS3, were increased significantly. In terms of the expression levels of non-canonical Wnt signalling-related molecules Wnt-4/-5A/-5B, FZD-2/-4/-6 were significantly increased in WB-F344 cells. This result indicated that M1 macrophages promoted the activation of the non-canonical Wnt signalling pathway, which promoted the differentiation of hepatic progenitor cells into myofibroblasts. WIF-1 had no significant effect on macrophage polarization but significantly decreased α-SMA mRNA expression in WB-F344 cells. YGJ significantly inhibited macrophage polarization into the M1 phenotype, significantly promoted the activation of canonical Wnt signalling, and inhibited the activation of non-canonical Wnt signalling. These effects of YGJ reduced the mRNA expression of α-SMA in WB-F344 cells *in vivo*. Taken together, these results suggest that Wnt signalling serves as a key ‘regulator’ in the cross-talk between macrophages and hepatic progenitor cells in rat liver fibrotic model induced by 2-AFF/CCl_4_. Likewise, YGJ may regulate macrophage polarization and Wnt signalling pathway mediated cross-talk between macrophages and hepatic progenitor cells.

## Conclusions

We found that in the rat liver fibrosis induced by 2-AAF/CCl_4_, YGJ inhibited macrophage M1 polarization, thereby reducing the release of non-canonical Wnt ligands. The activation of non-canonical Wnt signalling in hepatic progenitor cells was inhibited, and in turn, their differentiation into myofibroblasts was inhibited. This study not only provides further evidence for the anti-hepatic fibrosis effect of YGJ but also presents a scientific basis for the use of YGJ in the treatment of cirrhosis.

## Supplementary Material

Supplemental Material
