# Supplementary material for: Yiguanjian decoction inhibits macrophage M1 polarization and attenuates hepatic fibrosis induced by CCl4/2-AAF
Source: Pharm Biol. 2021 Aug 23;59(1):1148–58. doi: 10.1080/13880209.2021.1961820 (PMC8436970; doi:10.1080/13880209.2021.1961820)
Supplement: Supplemental Material [file IPHB_A_1961820_SM3621.docx]

Supplementary Material

**Chemical Analysis of Yiguanjian Decoction (YGJ) by UHPLC-Q-Orbitrap HRMS**

The chemical analysis of extract of YGJ was analyzed by using ultra-high-performance liquid chromatography-Q exactive hybrid quadrupole orbitrap high-resolution accurate mass spectrometric (UHPLC-Q-Orbitrap HRMS, Thermo Fisher Scientific Inc., Grand Island, NY, USA). The UHPLC was Thermo Scientific Dionex Ultimate 3000 and controlled by Chromeleon 7.2 Software. The cooling autosampler was set at 10°C and protected from light, and the column heater was set at 40°C. A Waters ACQUITY UPLC HSS T3 column (2.1 × 100 mm, 1.8 μm) was employed and the column temperature was set at 40°C. The mobile phase consisted of A (acetonitrile) and B (0.1% formic acid) at a flow rate of 0.4 mL·min−1 and eluted with gradient elution: 0-2 min (2% A), 2-12 min (2%-95% A), 12-14min (95% A), 14-15 min (2% A). The injection volume was 5 μL.

The mass spectrometer Q-Orbitrap system was connected to the UHPLC system via a heated electrospray ionization and controlled by Xcalibur 4.1 software that was used for data capture and analysis. The electrospray ionization source was operated and optimized in negative ionization mode. The optimized parameters of mass spectrometry were: capillary temperature: 325°C; sheath gas (N2) flow rate: 45 arbitrary units; auxiliary gas (N2) flow rate: 8 arbitrary units; sweep gas flow rate: 0 arbitrary units; spray voltage: 2.8 kV (negative); S-lens RF level: 50V; auxilliary gas heater temperature, 300°C; scan mode: full MS: scan range: 100–1500 m/z, the ions of target compounds catalpol ([M+HCOOH-H]- m/z 407.11840, characteristic component of Rehmannia glutinosa Libosch), verbascoside ([M -H]- m/z 623.19704, characteristic component of Rehmannia glutinosa Libosch), ferulic acid ([M-H]- m/z 193.04953, characteristic component of Angelica sinensis (Oliv.) Diels), and toosendanin ([M+HCOOH-H]- m/z 619.23851, characteristic component of seed of MeLia toosendan Sieb.et Zucc) were extracted for quantitative and qualitative analysis; maximum injection time (IT): 200 ms; scan resolution, 70,000 FWHM (m/z/s); automatic gain control (AGC) target: 1.0e6. The typical chromatographic fingerprints of extract of YGJ and mixture reference standards of catalpol, verbascoside, ferulic acid and toosendanin were deposited in Fig. X. The contents of targeted markers catalpol, verbascoside, ferulic acid and toosendanin in YGJ were determined as 946.61 μg/g, 54.06 μg/g, 89.00μg/g and 1.51 μg/g, respectively. The UPLC fingerprint of YGJ is illustrated in Supplementary .

**
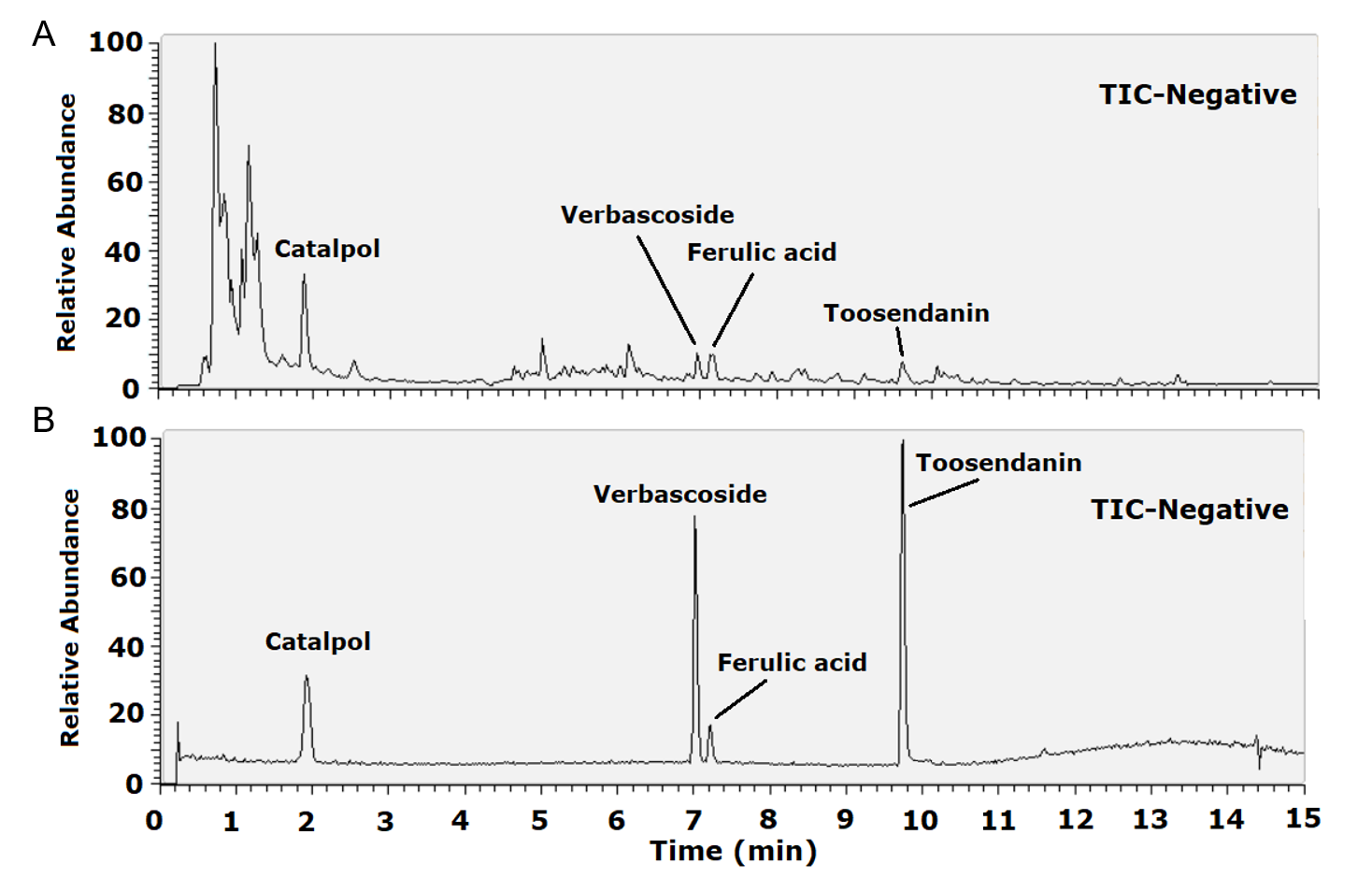
Supplementary Figure.** **Chemical analysis of YGJ by UHPLC-Q-Orbitrap HRMS.** (A) The fingerprint chromatograms of extract of YGJ. (B) The mixture reference standards in negative model.
